# Editorial: The Role of Immediate Early Genes in Neuropsychiatric Illness

**DOI:** 10.3389/fnbeh.2020.00016

**Published:** 2020-02-21

**Authors:** Amelia L. Gallitano

**Affiliations:** Departments of Basic Medical Sciences and Psychiatry, University of Arizona College of Medicine – Phoenix, Phoenix, AZ, United States

**Keywords:** immediate early gene, mental disorder, environment, stress, memory

The field of psychiatry lags behind other areas of medicine in not yet having identified a single gene that causes a mental illness (excluding neuro-developmental or neurodegenerative disorders such as autism spectrum disorders or dementias). This is due to the “complex genetics” that underlie these disorders. Specifically, *many* genetic variations across the genome influence risk for neuropsychiatric illnesses, no single one of which is responsible for a large percentage of cases, and non-genomic factors play a major role in their development. Together, these form the two major challenges that the field of psychiatry faces to identify the causes of mental health disorders: (1) how so many genes can influence risk for these illnesses, and (2) how environment interacts with predisposing genetic variations to result in neuropsychiatric illness.

A category of genes called immediate early genes (IEGs) are poised to answer both of these questions. IEGs are rapidly activated in the brain in response to neuronal activity which, itself, is triggered by environmental events. Many IEGs encode transcription factors, which regulate an array of target genes that carry out the cellular response to the stimulus. Thus, IEGs are positioned at the nexus between environmental stimuli, and the molecular events that can dictate long-term changes in the brain, including processes such as synaptic plasticity and memory formation. From this position, IEGs may determine both the “gene-environment” and “multiple-gene” influences on risk to develop psychiatric illness. The research, review, and hypothesis articles comprising this Research Topic explore “The Role of Immediate Early Genes in Neuropsychiatric Illness.”

One of the leading categories of environmental factors that increase risk for neuropsychiatric illnesses is stress. This term encompasses events ranging from *in utero* exposure to famine or infection, to stressful life events. Stress also activates expression of IEGs. In their Hypothesis/Review, Marballi and Gallitano describe the cascade of proteins activated in neurons in response to stress that culminate in expression of *Egr3*, a member of the Early Growth Response (*Egr*) family of IEG transcription factors. This stress-responsive “biological pathway” is essential for memory formation and synaptic plasticity. They point out that numerous genes encoding the proteins in this pathway have been associated with risk for schizophrenia, and present a model to explain how this pathway may represent a neuroprotective response to stress. Thus, variations that result in an insufficient activation of this protective stress-responsive pathway may result in the neuropathology that gives rise neuropsychiatric illness in individuals exposed to stress. However, an individual carrying the same susceptibility variations who is not exposed to significant stressors may not develop illness.

Once activated, IEGs mediate processes such as growth factor regulation, myelination, vascularization, synaptic plasticity, and memory and cognition. Notably, dysfunction in each of these areas has been implicated in mental illness pathogenesis. Two papers in the Research Topic address interactions between *Egr3* and the critical growth factor brain-derived neurotrophic factor (BDNF). In their review article, Pfaffenseller et al. integrate results from their group and others, showing that peripheral BDNF levels are reduced in patients experience mood disorders, with their recent findings indicating that *Egr3* is a master regulator of genes that are reduced in the prefrontal cortex of bipolar disorder patients. Prior studies showing that *Egr3* is activated downstream of BDNF, and suggesting that BDNF may play a beneficial role in bipolar disorder, lead them to propose a “feed-forward” model in which both BDNF and *Egr3* play protective roles, and are reduced in bipolar disorder.

The second of these articles reports the novel research finding that *Egr3* is required for the induction of hippocampal BDNF expression in response to electroconvulsive seizure, a known stimulus of BDNF expression Meyers et al. These results suggest that *Egr3* may regulate activity-dependent expression of BDNF, and support the model of a feed-forward pathway proposed by Pfaffenseller et al. but providing the evidence that *Egr3* is acting upstream, in addition to being activated downstream, of BDNF. The results of Meyers et al. provide a mechanism to suggest how dysfunction of *Egr3* may result in the BDNF deficiency found in bipolar disorder.

Other articles in the Research Topic address the role of IEGs in reward circuitry and addictive disorders. Manning et al. examine the role that IEGs play in stress-dependent remodeling of neural circuits involved in reward. Changes in the circuitry linking brain regions involved in pleasure, motivation, memory, decision-making and behavior is thought to influence mood disorders as well as addictive behaviors. After describing the cortico-basal ganglia reward network, they review the stress-responsive expression, and evidence for potential contributions to mood and addictive disorders, of a range of IEGs: *CREB, SRF, Egr1, Arc, NPAS4, Homer1a*, and the AP-1 Proteins c-fos, FosB/ΔFosB, and Jun. They conclude by posing key questions about the roles of IEGs that must be answered to advance our understanding of how these important genes may be influencing neuropsychiatric illnesses.

In their review article, Chandra and Lobo make the important point that IEGs are not simply markers of neuronal activity in response to processes but, in fact, play essential roles in the neurobiology underlying them. They focus on findings indicating that specific IEGs expressed in subsets of neurons in the striatum mediate cellular and molecular processes influencing plasticity and behavior that may mediate addiction to psychostimulants. The reviewed studies have relevance to neuropsychiatric disorders characterized by dysfunction in reward-seeking behaviors and motivational states as well as habitual behaviors.

In their research article, Muñiz et al. examine the role that caffeine plays in potentiating the effects of cocaine on reward-related learning in mice. They examined the expression of IEGs and dopamine receptor (DAR) subtypes in animals learning to associate a novel environment with drug exposure in the conditioned place preference paradigm. Their findings show that the combination of cocaine plus caffeine induced a set of IEG and DAR subtypes in the nucleus accumbens and prefrontal cortex that differ from the genes activated in response to cocaine alone. Their finding suggests that caffeine, the most widely used psychostimulant worldwide, may potentiate the effect of cocaine in reward-related memory formation.

Another group of articles address the contribution of IEGs to processes of learning and memory. Many IEGs have essential functions in memory formation and regulation of synaptic plasticity. Thus, defects in the functioning of IEGs could contribute to the cognitive symptoms characteristic of many neuropsychiatric illnesses. Duclot and Kabbaj provide a comprehensive review of the roles of a single IEG, *Egr1*, in the central nervous system. *Egr1* is an IEG transcription factor required for memory reconsolidation and the late phase of long-term potentiation (LTP), a form of synaptic plasticity (Jones et al., 2001). The authors systematically review the upstream processes that activate *Egr1*, as well as the downstream genes, pathways, and biological functions of *Egr1*. They conclude with a discussion of the role of *Egr1* in neuronal physiology, and how its dysfunction may contribute to neurological and psychiatric disorders.

In their review article, Gallo et al. address the critical role of numerous IEGs in learning, memory, and synaptic plasticity, pointing out how deficiencies in these processes may underlie the cognitive deficits characteristic of neuropsychiatric disorders. They expand their focus on *Egr1* to including the additional IEGs *c-fos* and *Arc*, covering the molecular actions of these genes in brain and their roles in learning and memory. With this framework, they then discuss the potential contribution that these IEGs may make in neuropsychiatric disorders ranging from mood disorders, to PTSD, to schizophrenia.

Datko et al. contribute an original research article in which they decipher the separate roles of the constitutively active form of the *Homer1* gene product from the IEG transcript, which encodes the protein *Homer1a*. The authors have previously reported that *Homer1a* is required for the establishment of cocaine-induced behavioral and neurochemical sensitization. In the current study of a mouse that selectively lacks *Homer1a*, they find that, rather unexpectedly, the IEG isoform is not required for spatial learning or conditioning in response to cocaine. This is in contrast to numerous other IEGs which are required for memory formation in some form.

Finally, two articles address the relationship between IEGs and the neurobiology and treatment of the psychotic disorder schizophrenia. Managò and Papaleo review the range of evidence from human and animal research studies suggesting that dysfunction of the IEG *Arc* may play a role in schizophrenia. The initial evidence arose from numerous genome-wide associations studies that identified genes that share the common feature of encoding proteins that interact with ARC. They further summarize the gene expression and association studies examining Arc, as well as findings from a family carrying a deletion that disrupts *ARC*, in addition to other genes, that has psychiatric and cognitive symptoms. The authors then employ the Research Domain Criteria (RDoC) as a framework for reviewing findings from the animal literature demonstrating functions of Arc in systems that are dysfunctional in neuropsychiatric illnesses. The authors complete their review with a summary of the roles that *Arc* plays in neurobiology, including in the dopaminergic and glutamatergic systems.

Finally, a comprehensive review by de Bartolomeis et al. addresses the clinically important question of how chronic use of antipsychotic medications causes long-term changes in the brain. While much of research examining the neurobiological effects of these medication has focused on acute changes, much less is known about the consequences of chronic treatment. IEGs have been used for decades as markers of neuronal activity. The authors review the literature examining brain regional activity following acute vs. chronic treatment with antipsychotic medications to examine effects on cognition, behavior, and the symptoms of psychosis. The authors suggest the intriguing possibility that these medication-induced changes in IEG expression may mediate the long-term neurobiological changes responsible for the therapeutic effects of antipsychotic medications.

Together these findings imply a critical role for IEGs, and the pathway of genes in which they function, in mediating the genetic and environmental influences on risk for mental illnesses. Ongoing genomics and post-mortem gene expression studies continue to reveal important findings suggesting that IEGs play a role in neuropsychiatric disorders. Studies in post-mortem brain tissue from schizophrenia patients indicate deficits in activity dependent IEGs, such as *EGR1*, and the genes that this transcription factor regulates (Ramaker et al., 2017). And studies from the ENCODE project have revealed that the network of genes that are reduced in schizophrenia patients' brains, but which correlate with antipsychotic medication treatment, are predominated by IEGs (Gandal et al., 2018). With the accelerating rate of discoveries being made with the coordinated effort of labs worldwide the field is sure to soon discover whether IEGs do, in fact, mediate the role of environmental stress on the risk to develop neuropsychiatric illnesses, and whether the genes they regulate account for a relevant proportion of the many genomic regions associated with these severe illnesses.

## Author Contributions

AG conceptualized and wrote the article.

### Conflict of Interest

The author declares that the research was conducted in the absence of any commercial or financial relationships that could be construed as a potential conflict of interest.
